# Plastid and mitochondrion genomic sequences from Arctic *Chlorella* sp. ArM0029B

**DOI:** 10.1186/1471-2164-15-286

**Published:** 2014-04-16

**Authors:** Haeyoung Jeong, Jong-Min Lim, Jihye Park, Young Mi Sim, Han-Gu Choi, Jungho Lee, Won-Joong Jeong

**Affiliations:** 1 Korea Research Institute of Bioscience and Biotechnology (KRIBB), 111 Gwahangno, Yuseong-gu, Daejeon 305-806, South Korea; 2 Green Plant Institute, #2-202 Biovalley, 89 Seoho-ro, Kwonseon-gu, Suwon, South Korea; 3 Korea Polar Research Institute, KIOST, 26 Songdomirae-ro, Yeonsu-gu, Incheon 406-840, South Korea

## Abstract

**Background:**

*Chorella* is the representative taxon of Chlorellales in Trebouxiophyceae, and its chloroplast (cp) genomic information has been thought to depend only on studies concerning *Chlorella vulgaris* and GenBank information of *C. variablis*. Mitochondrial (mt) genomic information regarding *Chlorella* is currently unavailable. To elucidate the evolution of organelle genomes and genetic information of *Chlorella*, we have sequenced and characterized the cp and mt genomes of Arctic *Chlorella sp*. ArM0029B.

**Results:**

The 119,989-bp cp genome lacking inverted repeats and 65,049-bp mt genome were sequenced. The ArM0029B cp genome contains 114 conserved genes, including 32 tRNA genes, 3 rRNA genes, and 79 genes encoding proteins. *Chlorella* cp genomes are highly rearranged except for a *Chlorella*-specific six-gene cluster, and the ArM0029B plastid resembles that of *Chlorella variabilis* except for a 15-kb gene cluster inversion. In the mt genome, 62 conserved genes, including 27 tRNA genes, 3 rRNA genes, and 32 genes encoding proteins were determined. The mt genome of ArM0029B is similar to that of the non-photosynthetic species *Prototheca* and *Heicosporidium*. The ArM0029B mt genome contains a group I intron, with an ORF containing two LAGLIDADG motifs, in *cox1*. The intronic ORF is shared by *C. vulgaris* and *Prototheca*. The phylogeny of the plastid genome reveals that ArM0029B showed a close relationship of *Chlorella* to *Parachlorella* and *Oocystis* within Chlorellales. The distribution of the *cox1* intron at 721 support membership in the order Chlorellales. Mitochondrial phylogenomic analyses, however, indicated that ArM0029B shows a greater affinity to MX-AZ01 and *Coccomyxa* than to the *Helicosporidium*-*Prototheca* clade, although the detailed phylogenetic relationships among the three taxa remain to be resolved.

**Conclusions:**

The plastid genome of ArM0029B is similar to that of *C. variabilis*. The mt sequence of ArM0029B is the first genome to be reported for *Chlorella.* Chloroplast genome phylogeny supports monophyly of the seven investigated members of Chlorellales. The presence of the *cox1* intron at 721 in all four investigated Chlorellales taxa indicates that the *cox1* intron had been introduced in early Chorellales as a *cis*-splice form and that the *cis*-splicing intron was inherited to recent Chlorellales and was recently *trans*-spliced in *Helicosporidium.*

## Background

Chloroplasts and mitochondria, organelles of higher plants and algae, play important roles in energy production, photosynthesis, and metabolite production required for maintaining life. Although numerous biological functions of both organelles rely considerably on proteins imported from nuclear encoded genes, understanding the organelle genome will provide a major impact in the fields of evolution, biology, and biotechnology.

Currently, many genome projects are in progress for green microalgae. To date, more than 20 organelle genomes have been completely sequenced in green microalgae [1]. Generally, chloroplasts and mitochondria in green algae have multiple copies of a single type of circular genome. In green algae, various plastid genome sizes have been reported: 37.7 kb in the non-photosynthetic alga *Helicosporidium* sp. and 203.8 kb in *Chlamydomonas reinhardtii*[2,3]. Plastid genomes in higher plants and green algae encode 88–138 genes [4,5]. Typical plastid genomes contain a large inverted repeat (IR) region with genes for rRNA, several tRNAs, and proteins. However, plastid genomes lacking an IR region also have been reported in some species [6,7]. The size of the mitochondrial (mt) genome varies among species: 6 kb in *Plasmodium* to 3,000 kb in the cucumber family [8,9]. The number of mt genes also varies: 5 genes in *Plasmodium* and about 100 genes in *Jakobid* flagellates [10].

*Chlorella* species, one of the best-known unicellular green algae, was studied in early research on photosynthesis [11] and is now used as a model and source for biotechnology and commercial applications such as use as a food additive, feed, and bioenergy source. The *Chlorella* genus belongs to Trebouxiophyceae, one of the Chlorophyte groups [12]. Trebouxiophyceae, found mostly in soil and freshwater, is a large algal group including *Chlorella*, *Oocystis*, *Parachlorella*, *Coccomyxa*, and *Helicosporidium.* The availability of organellar genomic information in Trebouxiophyceae, however, is very limited. Plastid genomes of seven species (*Chlorella vulgaris* C-27, *Chlorella variabilis* NC64A, *Coccomyxa sp*. C-169., Trebouxiophyceae *sp.* MX-AZ01, *Helicosporidium sp*., *Oocystis solitaria*, and *Parachlorella kessleri*) in Trebouxiophyceae have been sequenced, and they display a wide range of genome sizes, gene content, and intron content [13,14]. An IR region is missing in the plastid genome of *Chlorella vulgaris* C-27 [15] and *Chorella variabilis* NC64A (Accession no. NC_015359) but is detected in most of the Trebouxiophyceae (*Coccomyxa sp*., *Parachlorella kessleri*, and *Oocystis solitaria*) group. To date, the complete mt genome sequences have been reported in four trebouxiophycean algae, and they show a limited range of genome sizes, gene repertoires, and intron content. Two of them are non-photosynthetic relatives of *Chlorella*—*Prototheca wickerhamii*[16] and *Helicosporidium sp*. [17]. Two others are *Coccomyxa* sp. C-169 of Coccomyxaceae [18] and the unclassified Trebouxiophycean alga Trebouxiophyceae sp. MX-AZ01 [14]. However, the mt genome of *Chlorella* species remains unknown.

In the present study, we report the chloroplast (cp) and mt sequences of *Chlorella sp*. ArM0029B, which was isolated from drift ice in the Arctic region and has features of high lipid accumulation and fast growth at various temperatures [19]. The plastid genome of ArM0029B is similar to that of *C. variabilis* NC64A except for large inversions and fewer introns. The mt sequence of ArM0029B here is the first genome to be reported for *Chlorella*. We compared the *Chlorella sp*. ArM0029B organelle genome within Trebouxiophyceae and discussed cp phylogeny and *cox1* intron evolution. The unique features of both organelle genomes in *Chlorella sp.* ArM0029B presented here will provide an important insight into the evolution of organelle genomes within microalgal species and genetic information for biotechnology.

## Results and discussion

### Genomic organization and features of Arctic Chlorella sp. ArM0029B

**Figure 1:**
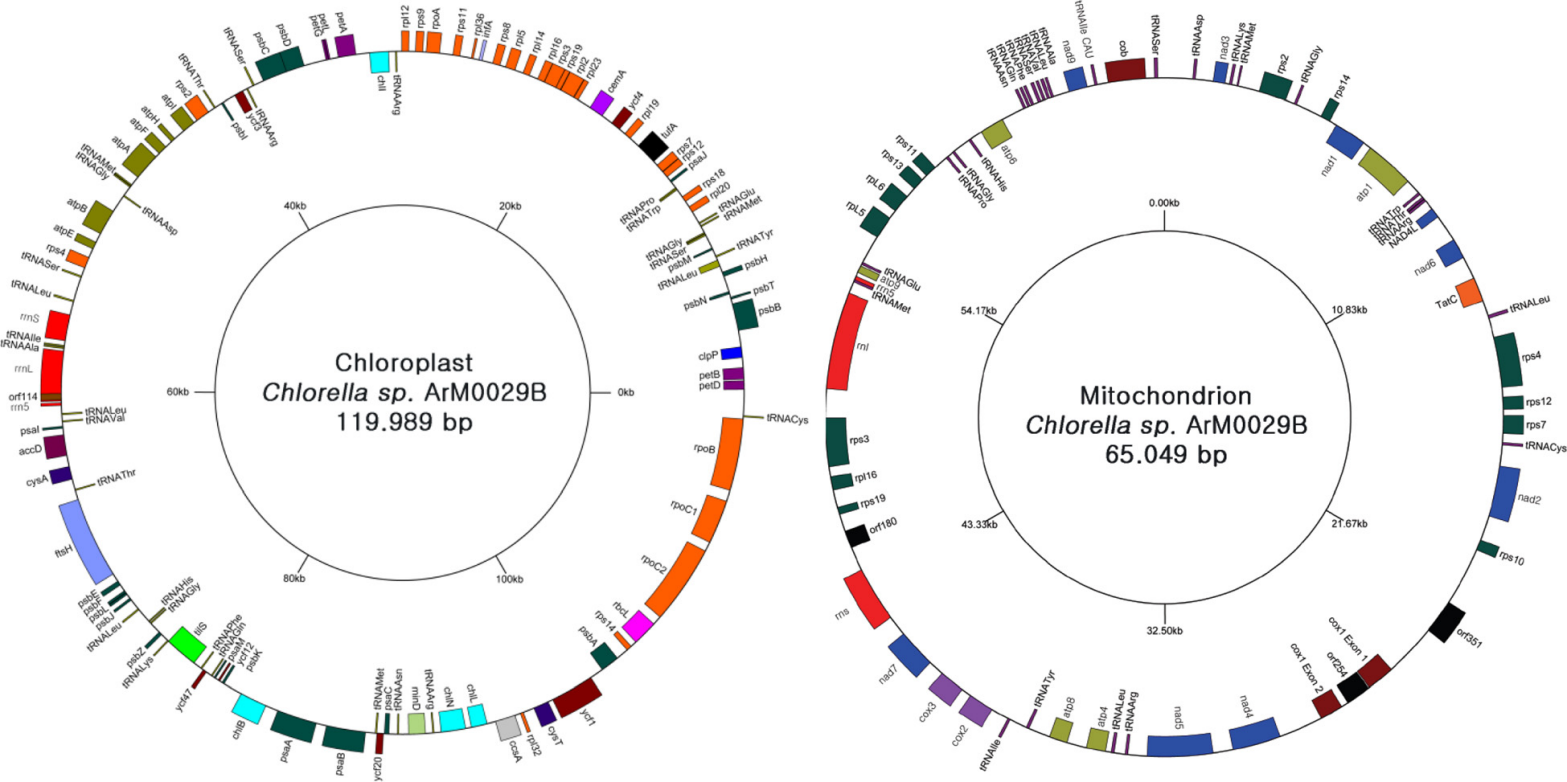
**Plastid and mt genomic maps of *Chlorella sp *. ArM0029B.**

The cp and mt genome sequences of ArM0029B were assembled as circular molecules of 119,989 bp and 65,049 bp, respectively (Figure 1). However, linear plastomes, concatenated pieces representing multiple plastomes (sometimes circular), and even branched forms were reported in many species [20,21]. The polymerase chain reaction (PCR) approach we used would not rule out linear, concatenated or branched structures of an organelle genome. Therefore, we cannot exclude other complex conformations of the organelle genome in ArM0029B. The cp genome of ArM0029B contains 114 genes excluding the non-conserved open reading frames (ORFs) encoding over 50 amino acids (Tables 1 and 2). BLASTP search against the NCBI NR database revealed that all of the 79 protein-coding genes were conserved (E value < 1E-6), while only five of them were conserved hypothetical proteins. We identified 71 additional ORFs using the Glimmer (see Additional file 1: Table S1), but they were not incorporated into the final gene set because only two of them showed homology to bacterial hypothtical proteins. ArM0029B does not carry large IRs in the plastid genome as well as *C. variabilis* NC64A, *C. vulgaris* C-27, *Coccomyxa* sp. C-169, and *Trebouxiophyceae* sp. MX-AZ01, indicating that all genes are present as a single copy. The general features and gene lists were compared (Tables 1 and 2). The overall GC content of the genome of *Chlorella* sp. ArM0029B is low (33.92%) similar to that of *C. variabilis* NC64A (33.93%) and *C. vulgaris* C-27 (31.6%) but in contrast to that of *Coccomyxa* sp. C-169 (50.71%) and *Trebouxiophyceae* sp. MX-AZ01 (56.25%). The length of all 114 conserved genes in the plastid genome of ArM0029B is 64,626 bp, and the genes account for a coding density of 53.8% of the total cp genome sequence. The latter value is the highest coding density among all reported *Chlorella* spp. to date. These results indicate that the cp genome of ArM0029B is more compact than those of the above comparable species. The mt genome of ArM0029B contains a total of 62 genes excluding the non-conserved ORFs among Trebouxioaceae (Table 1). Most of the ORFs encoding over 50 amino acids are not conserved based on NCBI BlAST search. The general features and gene list of the genome of ArM0029B were compared with four Trebouxiophyceae spp., including *Prototheca wickerhamii*, *Helicosporidium* sp., *Coccomyxa* sp. C-169, and *Trebouxiophyceae* sp. MX-AZ01 (Tables 1, 2, and 3). The gene number of the mt genome of ArM0029B is highest (62 genes) among the mt genomes of all sequenced species of Trebouxiophyceae. The overall GC content of the genome is low (28.5%) similar to that of *Prototheca wickerhamii* (25.8%) and *Helicosporidium* sp. (25.6%) but in contrast to that of two species with a high GC content, *Coccomyxa* sp. C-169 (53.8%) and *Trebouxiophyceae* sp. MX-AZ01 (53.4%). All 62 conserved genes on the mt DNA of ArM0029B cover 32,655 bp in length and account for a coding density of 50.2% of the total mt genome sequence, representing an intermediate range compared with all sequenced species of Trebouxiophyceae.

**Table 1:** General features of plastid and mt genomes in trebouxiophycean algal species

**Chloroplast**	***Chlorella*** **sp. ArM0029B**	***C. variabilis*** **NC64**	***C. vulgaris***	***Coccomyxa***	***Trebouxiophyceae*** **sp.**
Length	119989	124579	150613	175731	149707
AT contents (%)	66.08	66.07	68.44	49.29	43.75
# genes	114	114	115*	115	115
# conserved CDS	79	79	79	79	79
# tRNA	32	32	33	33	33
# rRNA	3	3	3	3	3
# introns	1	3	3	1	5
Coding density	53.8	50.5	38.7	39.9/40.8	47.7/48.5
CDS + strand	57	57	26	44	72
rRNA + srand	3	3	3	3	3
tRNA + strand	14	18	17	17	15
CDS - strand	22	22	51	35	8
rRNA - srand	0	0	0	0	0
tRNA - strand	18	14	16	15	17
Percent of +/−	64.9	68.4	40	55.6	77.39
**Mitochondria**	***Chlorella *** **sp. ArM0029B**	***Prototheca***	***Helicosporidium* sp.**	***Coccomyxa***	***Trebouxiophyceae*** **sp.**
Length	65049	55328	49343	65497	74423
AT contents (%)	71.5	74.2	74.4	46.8	46.6
# genes	62	61	60	59	56
# conserved CDS	32	30	32	30	30
# tRNA	27	26	25	26	23
# rRNA	3	3	3	3	3
# introns	I (1)	I (5)	I (4)	I (1) II (4)	I (7) II (4)
Coding density	50.2	59.8	64.99	48.38	42.64
CDS on + strand	18	13	9	29	29
CDS on - strand	14	18	23	1	1
tRNA/rRNA on + srand	17	12	10	28	26
tRNA/rRNA on - srand	13	17	18	1	0
Percent of +/−	56.5	59.3	68.3	96.7	98.2

*The asterisk indicates information without unnamed hypothetical ORFs in the cp sequence of *Chlorella vulgaris* C-27.

**Table 2:** Gene list of the plastid genome in trebouxiophycean algal species

	**ArM0029B**	**NC64**	**C. vulgaris**	**Coccomyxa**	**Trebouxiophyceae sp.**
					**MX-AZ01_NC_018569**
accD	o	o	o	o	o
atpA	o	o	o	o	o
atpB	o	o	o	o	o
atpE	o	o	o	o	o
atpF	o	o	o	o	o
atpH	o	o	o	o	o
atpI	o	o	o	o	o
ccsA	o	o	o	o	o
cemA	o	o	o	o	o
chlB	o	o	o	o	o
chlI	o	o	o	o	o
chlL	o	o	o*	o	o
chlN	o	o	o	o	o
clpP	o	o	o	o	o
cysA	o	o	o	o	o
cysT	o	o	o	o	o
ftsH	o	o	o	o	o*
infA	o	o	o	o	o
minD	o	o	o	o	o
petA	o	o	o	o	o
petB	o	o	o	o	o
petD	o	o	o	o	o
petG	o	o	o	o	o
petL	o	o	o	o	o
psaA	o	o	o	o	o
psaB	o	o	o	o	o
psaC	o	o	o	o	o
psaI	o	o	o	o	o
psaJ	o	o	o	o	o
psaM	o	o	o	o	o
psbA	o	o*	o	o	o*
psbB	o	o	o	o*	o
psbC	o	o*	o	o	o
psbD	o	o	o	o	o
psbE	o	o	o	o	o
psbF	o	o	o	o	o
psbH	o	o	o	o	o
psbI	o	o	o	o	o
psbJ	o	o	o	o	o
psbK	o	o	o	o	o
psbL	o	o	o	o	o
psbM	o	o	o	o	o
psbN	o	o	o	o	o
psbT	o	o	o	o	o
psbZ	o	o	o	o	o
rbcL	o	o	o	o	o
rpl12	o	o	o	o	o
rpl14	o	o	o	o	o
rpl16	o	o	o	o	o
rpl19	o	o	o	o	o
rpl2	o	o	o	o	o
rpl20	o	o	o	o	o
rpl23	o	o	o	o	o
rpl32	o	o	o	o	o
rpl36	o	o	o	o	o
rpl5	o	o	o	o	o
rpoA	o	o	o	o	o
rpoB	o	o	o	o	o
rpoC1	o	o	o	o	o
rpoC2	o	o	o	o	o
rps11	o	o	o	o	o
rps12	o	o	o	o	o
rps14	o	o	o	o	o
rps18	o	o	o	o	o
rps19	o	o	o	o	o
rps2	o	o	o	o	o
rps3	o	o	o	o	o
rps4	o	o	o	o	o
rps7	o	o	o	o	o
rps8	o	o	o	o	o
rps9	o	o	o	o	o
tilS	o	o	o	o+	o+
tufA	o	o	o	o	o
ycf1	o	o	o	o	o
ycf12	o	o	o	o	o
ycf20	o	o	o	o	o
ycf3	o	o	o	o	o
ycf4	o	o	o	o	o
ycf47	o	o	o	o	o
minE	*-*	*-*	o	*-*	*-*
trnA (UGC)	o	o	o	o	o
TrnC (GCA)	o	o	o	o	o
trnD (GUC)	o	o	o	o	o
trnE (UUC)	o	o	o	o	o
trnF (GAA)	o	o	o	o	o
trnG (UCC)	o	o	o	o	o
trnG (GCC)	oo	oo	oo	o	o
trnH (GUG)	o	o	o	o	o
trnI (GAU)	o	o	o	o	o
trnI (CAU)	o	o	o	o	o
trnK (UUU)	o	o	o	o	o
trnL (CAA)	o	o	o	o	o
trnL (GAG)	o	o	o	o	o
trnL (UAA)	o*	o*	o*	o	o
trnL (UAG)	o	o	o	o	o
trnM (CAU)	oo	oo	oo	oo	oo
trnN (GUU)	o	o	o	o	o
trnP (UGG)	o	o	o	o	o
trnQ (UUG)	o	o	o	o	o
trnR (ACG)	o	o	o	o	o
trnR (UCU)	o	o	o	o	o
trnR (CCG)	o	o	o	o	o
trnR (CCU)	*-*	*-*	*-*	o	o
trnS (GCU)	o	o	o	o	o
trnS (GGA)	o	o	o	o	o
trnS (UGA)	o	o	o	o	o
trnT (UGU)	o	o	o	o	o
trnT (GGU)	o	o	o	o	o
trnV (UAC)	o	o	oo	o	o
trnW (CCA)	o	o	o	o	o
trnY (GUA)	o	o	o	o	o
rrnL	o	o	o*	o	o***
rrnS	o	o	o	o	o
rrn5	o	o	o	o	o

*gene containing one intron; **gene containing two introns; ***gene containing three introns; oo, 2 copies; +, fragmented gene.

**Table 3:** **Distribution of the mt protein-coding gene and *trn *** **gene among trebouxiophycean algae and chlorophycean algae**

	**Trebouxiophyceae**					**Chlorophyceae**			
	**ArM0029B**	***Prototheca***	***Helicosporidium***	***Coccomyxa***	***MX-AZ01***	***Scenedesmus***	***Dunaliella***	***Gonium***	***Chlamydomonas***
atp1	o	o	o	o	o	*-*	*-*	*-*	*-*
atp4	o	*-*	o	o	o	*-*	*-*	*-*	*-*
atp6	o	o	o	o	o	o	*-*	*-*	*-*
atp8	o	o	o	o	o	*-*	*-*	*-*	*-*
atp9	o	o	o	o	o	o	*-*	*-*	*-*
cob	o	o	o	o	o	o	o	o	o
cox1	o	o	o	o	o	o	o	o	o
cox2	o	o	o	o	o	o	*-*	*-*	*-*
cox3	o	o	o	o	o	o	*-*	*-*	*-*
nad1	o	o	o	o	o	o	o	o	o
nad2	o	o	o	o	o	o	o	o	o
nad3	o	o	o	o	o	o	*-*	*-*	*-*
nad4	o	o	o	o	o	o	o	o	o
nad4L	o	o	o	o	o	o	*-*	*-*	*-*
nad5	o	o	o	o	o	o	o	o	o
nad6	o	o	o	o	o	o	o	o	o
nad7	o	o	o	o	o	*-*	*-*	*-*	*-*
nad9	o	o	o	o	o	*-*	*-*	*-*	*-*
rpl5	o	*-*	o	o	o	*-*	*-*	*-*	*-*
rpl6	o	o	o	*-*	*-*	*-*	*-*	*-*	*-*
rpl16	o	o	o	o	o	*-*	*-*	*-*	*-*
rps2	o	o	o	o	o	*-*	*-*	*-*	*-*
rps3	o	o	o	o	o	*-*	*-*	*-*	*-*
rps4	o	o	o	o	o	*-*	*-*	*-*	*-*
rps7	o	o	o	o	o	*-*	*-*	*-*	*-*
rps10	o	o	o	o	o	*-*	*-*	*-*	*-*
rps11	o	o	o	*-*	*-*	*-*	*-*	*-*	*-*
rps12	o	o	o	o	o	*-*	*-*	*-*	*-*
rps13	o	o	o	o	o	*-*	*-*	*-*	*-*
rps14	o	o	o	o	o	*-*	*-*	*-*	*-*
rps19	o	o	o	o	o	*-*	*-*	*-*	*-*
tatC	o	o	o	o	o	*-*	*-*	*-*	*-*
size	65,049	55,328	49,343	65,497	74,423	42,781	28,331	15,993	15,758
**tRNAs**	**Trebouxiophyceae**					**Chlorophyceae**			
	**ArM0029B**	***Prototheca***	***Helicosporidium***	***Coccomyxa***	***MX-AZ01***	***Scenedesmus***	***Dunaliella***	***Gonium***	***Chlamydomonas***
trnA (UGC)	o	o	o	o	o	o	_	_	_
trnC (GCA)	o	o	o	o	o	o	_	_	_
trnD (GUC)	o	o	o	o	o	o	_	_	_
trnE (UUC)	o	o	o	o	o	o	_	_	_
trnF (GAA)	o	o	o	o	o	o	_	_	_
trnG (GCC)	o	o	_	o	o	_	_	_	_
trnG (UCC)	o	o	o	o	_	o	_	_	_
trnH (GUG)	o	o	o	o*	o*	o	_	_	_
trnI (CAU)	o	o	o	o	o	_	_	_	_
trnI (GAU)	o	o	o	o	o	o	_	_	_
trnI (UAU)	_	_	_	_	_	o	_	_	_
trnK (UUU)	o	o	o	o	o	o	_	_	_
trnL (AAG)	_	_	_	_	_	o	_	_	_
trnL (CAA)	o	_	_	o	o	_	_	_	_
trnL (CAG)	_	_	_	_	_	o	_	_	_
trnL (UAA)	o	o	o	o	o	_	_	_	_
trnL (UAG)	o	o	o	o	o	_	_	_	_
trnM (CAU)	oo	oo	oo	oo	o	oo	o	oo	o
trnN (GUU)	o	o	o	o	o	o	_	_	_
trnP (UGG)	o	o	o	o	_	o	_	_	_
trnQ (UUG)	o	o	o	o	o	o	o	o	o
trnR (ACG)	o	o	o	o	_	o	_	_	_
trnR (CCU)	_	_	_	_	_	o	_	_	_
trnR (UCU)	o	o	o	o	o	o	_	_	_
trnS (GCU)	o	o	o	o*	o*	o	_	_	_
trnS (GGA)	_	_	_	_	_	o	_	_	_
trnS (UGA)	o	o	o	o*	o*	_	_	_	_
trnT (UGU)	o	o	o	_	_	_	_	_	_
trnV (UAC)	o	o	o	o	o	o	_	_	_
trnW (CCA)	o	o	o	o*	o*	o	o	o	o
trnW (CUA)	_	_	_	_	_	o	_	_	_
trnY (GUA)	o	o	o	o	o	o	_	_	_
Total number	27	26	25	26	23	27	3	4	3

*gene containing intron; oo, 2 copies.

In the cp genome, with 74 (64.9%) conserved genes occupying one strand and 40 genes occupying the other strand, the gene distribution over the two DNA strands of ArM0029B cp genome is biased (Figure 1, Table 1). The gene contents in one strand were detected to be 68.4%, 40.5%, and 55.6% in the cp genome of NC64A, *C. vulgaris* C-27 and *Coccomyxa* C-169, respectively. These results indicate that gene distribution between the two strands of the cp genome is biased to some degree but relatively even in contrast to one of the mt genomes. In the mt genome of ArM0029B, 35 conserved genes occupy one strand, and 27 genes occupy the other strand, indicating that the genes are evenly (56.5:43.5) distributed in both strands of the ArM0029 mt genome (Table 1). The other two species, *Prototheca wickerhamii* and *Heicosporidium* sp., showed more biased occupation of the genes in one strand (59.3% and 68.3%, respectively) than *Chlorella* sp. ArM0029B. Furthermore, *Coccomyxa* and MX-AZ01 displayed a drastic biased distribution of the mt genes in one strand (96.7% and 98.2%, respectively).

### Gene content and rearrangement of the cp genome

**Figure 2:**
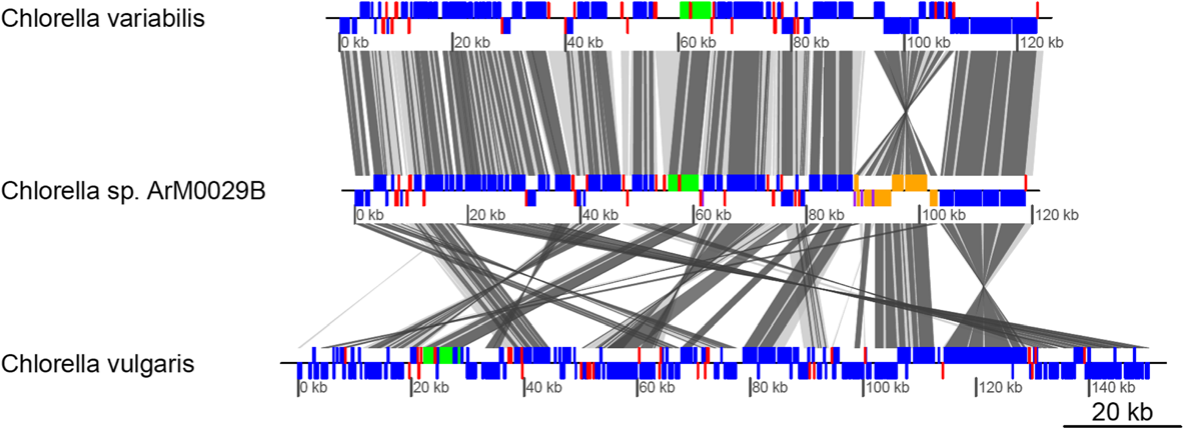
**Comparison of the cp genomes of *C. variabilis*, *Chlorella*sp. ArM0029B, and *C. vulgaris*.** Thick bars represent protein-coding genes (blue), rRNA genes (green), and tRNA genes (red). Genes in the inverted region were highlighted in orange (protein-coding genes) and purple (tRNA genes) boxes in ArM0029B. LCBs and conserved genes are connected by light and dark gray rhomboids, respectively.

The plastid genome of ArM0029B contains 79 genes encoding proteins, 32 tRNA genes, and 3 rRNA genes similar to that of *C. variabilis* NC64A (Table 2). The ArM0029B plastid gene repertoire differs from that of *C. variabilis* NC64A except for the absence of pseudogenes similar to *chlL* and an intronic endonuclease in the *psbC* gene, and from *C. vulgaris* C-27 by the absence of tRNA-Val (UAC) and the *minE* homolog. AM0029B has a small cp genome among species although it has a similar number of genes to *C. variabilis* NC64A, *C. vulgaris* C-27, *Coccomyxa* sp. C-169, and *Trebouxiophyceae* sp. MX-AZ01 (Tables 1 and 2). The compactness of the cp genome of ArM0029B is due to a short intergenic sequence and fewer introns. The conserved gene order and rearrangement of cp genomes among ArM0029B, *C. variabilis*, and *C. vulgaris* were compared in Figure 2. The gene order in the plastid genome of *Chlorella* sp. ArM0029B is very similar to that of *C. variabilis* NC64A. Rearrangement of genomes between ArM0029B and *C. variabilis* was found in two regions; *trnV* and the 15-kb gene cluster, including “*trnI-ycf20-psaC-trnN-minD-trnR1-chlN-chlL-ccsA-rpl32-cysT-ycf1-psbA*”, are present in inverse orientation between *Chlorella* sp. ArM0029B and *C. variabilis* NC64A (Figure 2 and see Additional file 2: Figure S1). Marked rearrangement of gene clusters was detected between ArM0029B and *C. vulgaris*. Many gene clusters conserved in green algae [22] are also conserved in *Chlorella sp.* ArM0029B (Figure 2). Interestingly, the gene order of “*trnC-rpoB-rpoC1-rpoC2-rbcL-rps14*” is well conserved between ArM0029B and two *Chlorella* spp., *C. vulgaris* and *C. variabilis* (see Additional file 3: Figure S2) but not in related species *Coccomyxa* sp. C-169 and *Trebouxiophyceae* sp. MX-AZ01, suggesting that the gene order is well conserved and may be specific to *Chlorella* species. The order of *psbD* and *psbC* genes are conserved and closely linked in all sequenced Trebouxiophyceae. Interestingly, the 5′ coding region of the *psbC* gene seemed to be overlapped with the 3′ coding region of *psbD* on the same strand in ArM0029B. This phenomenon of two genes overlapping occurs frequently in the genomes of viruses, prokaryotes, mitochondria, and eukaryotes, including humans [23-25]. The overlap of *psbD* and *psbC* seemed to exist in all of the Trebouxiophyceae sequenced except for *Helicosporidium sp.*, which lacks *psbD* and *psbC* in the plastid genome. The *psbC* gene in *Coccomyxa* sp. C-169 and *Trebouxiophyceae* sp. MX-AZ01 was annotated with Gly as a starting amino acid, resulting in separation of *psbC* from *psbD*. Possible Gly start codons of *psbC* are also found a few bases after *psbD* in all sequenced Trebouxiophyceae. However, the ATG or GTG start codon is also found in the 3′ coding region of *psbD* in those species. In other class of viridiplantae, *Oltmannsiellopsis viridis* and *Pseudendoclonium akinetum* of Ulvophyceae, *Nephroselmis olivacea* in Prasinophytes, and *Mesostigma viride* in Charophyceae also share the same feature of overlapping of *psbD*-*psbC* or a GTG start codon of *psbC* without overlap with *psbD*. However, in the case of *psbC* separated clearly from *psbD* such as in *C. reinhardtii* and *Senedesmus*, the N-terminal amino acid sequence of *psbC* is *Met-Glu-Thr-Leu-Phe-Asn*-Gly-Thr(Ser). The italic amino acids are well conserved and are encoded in all overlapped sequences of the above species, indicating that all linked genes of *psbD* and *psbC* may be overlapped in the same manner.

### Gene content of the mt genome

The mt genome of ArM0029B contains 32 mt protein coding genes, 27 tRNA genes, and 3 rRNA genes (Tables 1 and 3). The 32 protein-coding genes include 4 *atp* genes, 3 *cox* genes, 9 *nad* genes, 13 ribosomal protein genes, and *cob* and *tatC* genes. *Helicosporidium*, another trebouxiophycean alga, also has the same content of protein-coding genes. Three other trebouxiophycean algae, *Prototheca*, *Coccomyxa*, and MX-AZ01, have only 30 among 32 protein-coding genes of ArM0029B and *Helicosporidium* (Table 3). Two ribosomal protein genes, *rpl6* and *rps11*, are absent in *Coccomyxa* and MX-AZ01. *Prototheca* lacks two genes, *atp4* and *rpl5*. It is assumed that the genes in the three taxa were recently lost in the lineage of Trebouxiophyceae, possibly nuclear transferred. Compared with trebouxiophycean algae, the chlorophycean algae, including *Scenedesmus*, *Dunaliella*, *Gonium*, *and Chlamydomonas* do not have any ribosomal protein gene and *tatC*, which are found in Trebouxiophyceae (Table 3). *Scenedesmus* has the largest content of protein-coding genes among the chlorophycean algae with 13 protein-coding genes, all shared by trebouxiophycean algae. The 13 protein-coding genes include 2 *atp* genes, 3 *cox* genes, 7 *nad* genes, and a *cob* gene. Among the 13 genes, 6 are absent in the other three chlorophycean algae, *Dunaliella*, *Gonium*, *and Chlamydomonas*. The three chlorophycean algae contain the other seven protein-coding genes, including *cob*, *cox1*, *nad1*, *nad2*, *nad4*, *nad5*, and *nad6*. The ArM0029B mt genome contains 27 tRNA genes, the largest in number among trebouxiophycean algae (Table 3). The tRNA gene content of other trebouxiophycean algae ranged from 23 to 26. Both *Coccomyxa* and MX-AZ01 share introns in four tRNA genes. In Chlorophyceae, although *Scenedesmus* has 27 tRNA genes, *Dunaliella*, *Gonium*, *and Chlamydomonas* have only three types of tRNA genes, *trnM*, *trnQ*, and *trnW*. Additional file 4: Figure S3 shows the characterization of the mt tRNA genes of *Chlorella sp*. ArM0029B. Amongst trebouxiophycean algae, *Chlorella sp*. ArM0029B has the largest number of tRNA genes, and the secondary structure of tRNA genes in trebouxiophycean algal mitochondria is unknown. Thus far, the *Chlorella sp*. ArM0029B is known to contain the largest gene content of the mt genomes of Trebouxiophyceae.

Analysis of the conserved gene cluster in the mt genome is difficult because of limited information regarding mt genomes in trebouxiophycean algae. The mt genome of *Chlorella* species was not reported except for ArM0029B of this study. It has been reported that the overall gene order in mt genomes is conserved between the non-photosynthetic group “*Prototheca wickerhamii* and *Helicosporidium* sp” and the high-GC content group “*Coccomyxa* sp. C-169 and *Trebouxiophyceae* sp. MX-AZ01”, respectively. [14,17]. The overall gene order on the mt genome of Chlorella sp. ArM0029B is not conserved with any member of the non-photosynthetic group or high-GC group. Nevertheless, the gene order for “*trnS-trnV-trnL*” and “*trnY-atp8-atp4*” is well conserved in all five species of Trebouxiophyceae.

### Introns in the organellar genomes of ArM0029B

**Figure 3:**
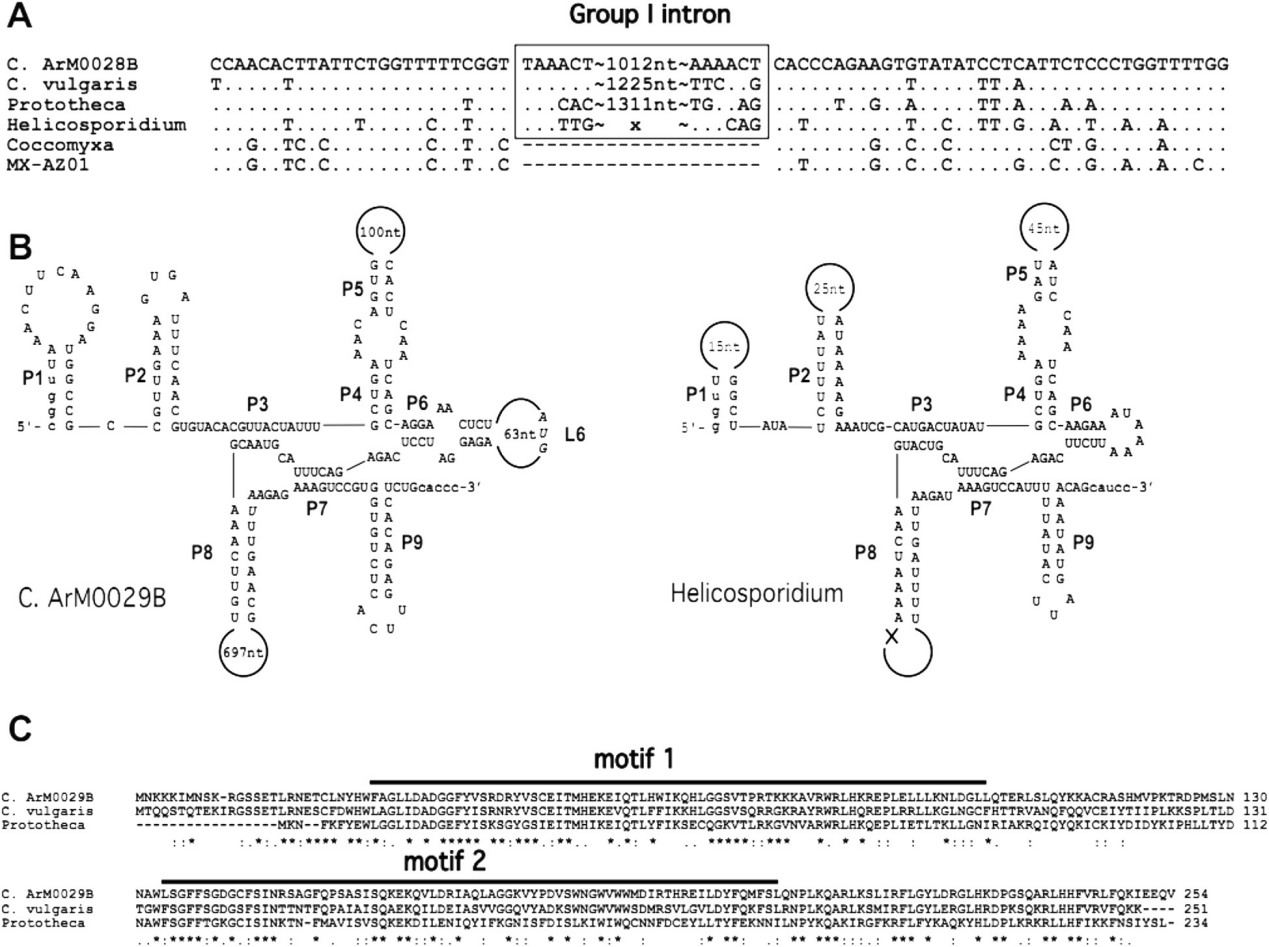
**The mt*cox1 *intron of *C. *ArM0028B and its distribution among trebouxiophycean algae. A**. The exon and intron border sequences. **B**. Secondary structures of the ArM0028B *cis*-splicing intron and *Helicosporidium trans*-splicing intron. **C**. Comparison of the intronic ORF with two LAGLIDADG motifs.

Two group I introns are found in the organellar genomes of *Chlorella sp*. ArM0029B. One resides in *trnL* (UAA) of the cp genome, and the other is located in *cox1* of the mt genome. The *trnL* (UAA) group I intron of cyanobacterial origin is an ancient self-splicing group I intron in the plastid genome that is rarely lost in some taxa [26,27]. The ArM0029B mt genome has the intron between bases 720 and 721 of *cox1*. Among trebouxiophycean algae, the intron with the same insertion site is also found in *C. vulgaris*, *Prototheca*, and *Helicosporidium* but not in *Coccomyxa* and MX-AZ01 (Figure 3A). The *Chlorella sp*. ArM0029B intron is a *cis*-splicing intron and has an ORF starting at Loop 6 (L6) and ending at P8-P7 (Figure 3B). The ORF has two LAGLIDADG endonuclease motifs. The endonuclease-like ORF of the group I intron is known to have two LAGLIDADG motifs [28]. The ORF with two LAGLIDADG motifs is also found in the same intron of *C. vulgaris* and *Prototheca* (Figure 3C). Unlike *Chlorella* and *Prototheca*, *Helicosporidium* has a *trans*-splicing intron without an ORF. As shown in Figure 3B, the dis-connection of the intron in *Helicosporidium* occurs at loop 8, which contains the ORF, assuming that the *trans*-splicing intron of *Helicosporidium* might be derived from *cis*-splicing by genomic rearrangement, followed by loss of the ORF. Compared with the ArM0029B intron, other related species in Trebouxiophyceae contain 3–11 introns in two to six genes of their mt genomes (Table 1), indicating that the mt genome of ArM0029B has the smallest number of introns among those reported in Trebouxiophyceae. An intron or introns split the *cox1* gene into two exons in ArM0029B, four exons in *Prototheca wickerhamii*, three exons in *Helicosporidium* sp., and two exons with different cognate sites in *Trebouxiophyceae* sp. MX-AZ01, and no intron was found in *Coccomyxa* sp. C-169. The intron distribution on the plastid genome in ArM0029B is different from that of other trebouxiophycean algae: three introns in *trnL*3, *psbA*, and *psbC* of *C. variabilis* NC64A, three introns in *trnL*3, *rrnL*, and *chlL* of *C. vulgaris*, one intron in *psbB* of *Coccomyxa* sp. C-169, and five introns in *ftsH*, *psbA*, and *rrnL* of *Trebouxiophyceae* sp. MX-AZ01 (Tables 1 and 2). Fewer introns, the lack of pseudogenes, and shorter intergenic regions contributed to the more compact plastid genome of ArM0029B than that of *C. variabilis* NC64A and *C. vulgaris* C-27.

### Phylogenetic affinity of ArM0029B to other trebouxiophycean algae

**Figure 4:**
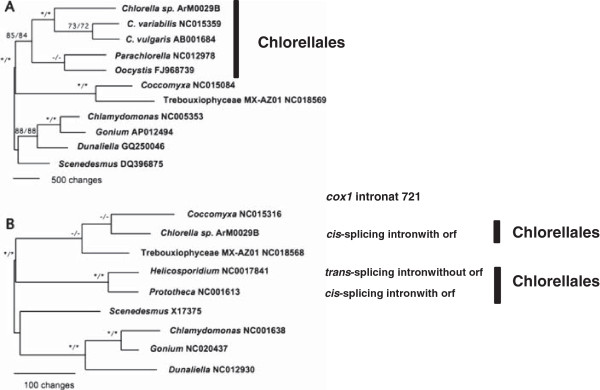
**Single maximum parsimonious (MP) trees. A**. The plastid MP tree from DNA sequences of *rbcL* and six photosystem genes. **B**. The mt MP tree from translated amino acid sequences of seven protein-coding genes. The distribution of *cox1* intron at 721, its splicing form, and presence/absence of its orf were denoted. */*: 100% bootstrap support/100% jackknife support. −/−: bootstrap and jackknife not supported.

Phylogenetic relationships among seven trebouxiophycean algal plastids were investigated using the aligned 10,938-base DNA sequence of six large photosystem genes—*psaA*, *psaB*, *psbA*, *psbB*, *psbC*, and *psbD*—and *rbcL*, which are widely used for phylogenetic studies [29-31]. Phylogenetic analysis outgrouped by four chlorophycean algae produced a single plastid maximum parsimonious (MP) tree (Figure 4A), and NJ and ML analyses showed a single tree with the similar topology (see Additional file 5: Figure S4). Four chlorophycean algae and six trebouxiophycean algae are separated into two sister clades with 100% bootstrap/Jackknife supports. Trebouxiophycean algae are separated into two clades: the *Coccomyxa*-[MX-AZ01] clade with 100% bootstrap/Jackknife supports and the *Chlorella-Parachlorella-Oocystis* clade with 84–85% bootstrap/Jackknife supports. Within a *Chlorella-Parachlorella-Oocystis* clade, ArM0029B-*Chlorella* 2 spp. formed a clade with 100% bootstrap/Jackknife support, but *Parachlorella* and *Oocystis* were clustered without bootstrap/Jackknife supports. The distance matrix of the aligned 10,938-base DNA sequence among seven trebouxiophycean algae is shown in Additional file 6: Table S2. The distance ranged from 9.193% to 10.299% between *Chlorella* species and ranged from 14.071% to 26.705% among Trebouxiophycean genera. The distance between ArM0029B and *C. variabilis* (9.193%) is smaller than the distance between ArM0029B and *C. vulgaris* C-27 (10.299%) or the distance between *C vulgaris* C-27 and *C. variabilis* (9.403%), indicating the close relationships of ArM0029B to *Chlorella variabilis*. The closer genus to *Chlorella spp*. was *Parachlorella* with 14.071 ~ 14.585% distance and *Oocystis* with 15.911 ~ 16.435% distance. Among other genera, *Parachlorella* and *Oocystis* had a 14.959% distance, and *Coccomixa* and MX-AZ01 had a 16.901% distance. Except for the genera discussed above, over 20% distance was detected among Trebouxiophycean genera. The results indicate that ArM0029B belongs to the genus *Chlorella* along with *C. vulgaris* and *C. variabilis* and that *C. variabilis* is the closer taxon to ArM0029B.The mt genome-based phylogenetic relationships among five trebouxiophycean algae were also analyzed using the translated amino acids sequences of seven genes, *cob*, *cox1*, *nad1*, *nad2*, *nad4*, *nad5*, and *nad6*, which are shared by trebouxiophycean and chlorophycean algal mitochondria. Phylogenetic analysis outgrouped by four chlorophycean algae produced a single MP tree (Figure 4B) and a single NJ tree with the same topology (see Additional file 5: Figure S4C). Four chlorophycean algae and five trebouxiophycean algae are separated into two sister clades with 100% bootstrap/Jackknife supports. The five-trebouxiophycean algae MP tree contained one clade, a *Helicosporidium*-*Prototheca* clade with 100% bootstrap/Jackknife support and three isolated taxa—ArM0029B, MX-AZ01, and *Coccomyxa*. Although the three taxa were clustered, the cluster was weakly bootstrap/Jackknife (65%/67%) supported. Although ArM0029B formed a clade with MX-AZ01 and *Coccomyxa* in MP and NJ trees, the mt phylogenomic affinity of ArM0029B to other trebouxiophycean algae remains to be investigated because of limited information of available mt genomes in trebouxiophycean algae. *Scenedesmus*, which contains the largest number of genes among Chlorophyceae, has ancient mt characteristics among green algae and is explained as a basal group in phylogenetic analysis. Green algae have lost many mt genes via gene transfer to the nucleus. ArM0029B contains more mt genes than other sequenced trebouxiophycean algae to date, suggesting that it may show ancient characteristics of its mt genome among trebouxiophycean algae. However, we cannot exclude the possibility of new integration of genes into the ancient-type trebouxiophycean alga with fewer genes in the mt genome.

### The Chlorellales in the phylogenies of chloroplasts and mitochondria and the meaning of cox1 intron at base position 721 of the cox1 gene

The Chlorellales is a green algal group lacking flagella whose members include *Chlorella*, *Parachlorella*, *Oocystis*, *Prototheca*, and *Helicosporidium. Chlorella* and *Parachlorella* inhabit in freshwater, marine, or land in the coccoidal form [32]. *Prototheca* and *Helicosporidium* are non-photosynthetic and parasitic coccoids. Phylogenetic relationships within the Chlorellales have been well studied, and nuclear and cp gene data have provided evidence that the Oocystaceae, including the semi-colonial *Oocystis*, form an early diverging clade within the Chlorellales [1]. Our cp genome phylogeny is congruent to those in previous reports and shows the formation of a strong clade containing *Chlorella*, *Parachlorella*, *Oocystis*, *Prototheca,* and *Helicosporidium*. The *cox1* intron at base position 721 in the *cox1* gene of the mt genome is found only in the members of Chlorellales in Trebouxiophyceae—i.e., *Prototheca*, *Helicosporidium*, *Chlorella vulgaris* and *Chlorella sp.* ArM0029B. The *cox1* intron distribution supporting Chlorellales does not agree with mt genome phylogeny. The occurrence of the *cox1* intron in free-living Arctic *Chlorella sp.* ArM0029B and *Chlorella vulgaris*, as well as in the parasitic coccoids *Prototheca* and *Helicosporidium*, indicates that the intron with the same origin had been introduced in early Chlorellales and that the *trans*-splicing of the intron occurred after the divergence of *Prototheca* and *Helicosporidium* in the parasitic coccoid clade. Limited taxon sampling, high variation of mt genes, and possible lateral gene transfer from other taxa might affect the topology of the phylogenetic tree in the present study. Increasing members of representative taxa in trebouxiophycean algae would help to improve the understanding of its evolution.

## Conclusions

Organelle functions play an important role in maintaining an organism’s life, including energy production, photosynthesis, and metabolite biosynthesis. The chloroplast is an organelle for fatty acid/lipid biosynthesis, and the mitochondrion is an organelle for fatty acid/lipid degradation. Recently, oil-producing microalgae have been studied intensively for genetic improvement, including genomics and genetic engineering. *Chlorella* is an important microalgae for oil production [33]. ArM0029B is a *Chlorella* sp. originated from the Arctic region, which have features of fast growth at various temperatures and a high oil-accumulating trait [19].

Here, we report the 119,989-bp cp genome and 65,049-bp mt genome of Arctic *Chlorella* sp. ArM0029B. The plastid genome of ArM0029B lacking a large IR is close to *C. variabilis* NC64A: both species displayed the same content of conserved genes and almost the same gene order. However, large rearrangements are also found between ArM0029B and *C. variabilis* NC64A by inversion of a 15-kb gene cluster. Major structural changes were detected in introns and tRNAs in ArM0029B compared with related species of Trebouxiophyceae. The mt genome of ArM0029B contains the largest number of genes (62 genes) and smallest number of introns (one intron in *cox1*) among trebouxiophycean algae. Detailed information regarding the secondary structure of the tRNA genes would be obtained in a *Chlorella* mt genome study. Two group I introns were found in ArM0029B: a self-splicing intron in *trnL* (UAA) of the cp genome and another intron in *cox1* of the mt genome containing an ORF encoding an endonuclease with double motifs of LAGLIDADG. Phylogenetic analysis of cp genomes suggests that three *Chlorella* species belong to a monophyletic group, and ArM0029B belongs to the genus *Chlorella*. The phylogenetic analysis of mt genomes with limited information of the available mt genome in Trebouxiophyceae could not determine the closest mt genome of ArM0029B among the four trebouxiophycean algae. The lowest number of introns in the organelle genome of ArM0029B among *Chlorella* spp. may be due to the limited chance of intron spreading and invasion by the isolation in the Artic environment from other related taxa. Based on the gene content, the ArM0029B organelle genomes seem to have ancient organelle characteristics with many genes and fewer introns gene in both genomes.

In the present study, cp genome phylogeny supports monophyly of the seven investigated members of Chlorellales, including three *Chlorella spp.*, *Parachlorella*, *Oocystis*, *Prototheca*, and *Helicosporidium*. The intron distribution at base position 721 of the *cox1* gene occurs in all four investigated Chlorellales taxa—*Chlorella sp.* ArM0029B, *Chlorella vulgaris*, *Prototheca*, and *Helicosporidium—*assuming that a common ancestor of the Chorellales might display the *cox1* intron as a *cis*-splice form and that the *cis*-splicing intron was recently *trans*-spliced in *Helicosporidium.* When more mt genomic information is available, we will have better understanding of the mt genome phylogeny of the trebouxiophycean algae.

The unique features of *Chlorella sp*. ArM0029B organelle genomes presented here will provide important information to understand organellar genome evolution, including introns, gene rearrangement, and structural changes of plastids and mt genomes among species in Trebouxiophyceae and green algae.

## Methods

### Strain and culture conditions

*Chlorella* sp. ArM0029B [19] was maintained on solidified TAP medium [34] and cultured in liquid medium for analysis at 25°C with 200 rpm shaking under constant white light (40 μmol m^−2^ s^−1^).

### Sequencing, assembly, and annotation of the ArM0029B organelle genomes

The ArM0029B organelle genomes were sequenced as part of the ArM0029B genome project (funded by Advanced Biomass R&D Center) using an Illumina HiSeq 2000-based whole-genome shotgun sequencing approach. The organelle sequences were obtained using the CLC Genomics Workbench version 5.5. Two large contigs (65.049 kb and 120.090 kb) with the highest average read coverages (19,505 and 7,485, respectively) were identified; the contigs displayed low GC content compared with the high GC content of the nuclear genome. Circular structures of each replicon were confirmed by polymerase chain reaction (PCR) amplification at their ends and by joining of Sanger sequence reads derived from the amplicons. The assemblies were further verified by examining paired-end distance and depth after re-mapping reads on the contig sequences. The BLAST searches of two large contigs were verified to plastid and mt genomes, respectively. For gene annotation of organelle genomes, ORFs encoding 50 amino acids or longer were identified and searched against a known protein database (NR). Genes encoding proteins homologous to known short cp peptides were manually identified. Glimmer (ver. 3.02) was used to predict additional putative protein-coding genes [35]. tRNA and rRNA genes were respectively detected using ARAGORN [36] and RNAmmer 1.2. The *rrn5* in the mt genome was detected based on BLAST search and the 5S rRNA data bank [37]. The complete sequences of the ArM0029B chloroplast and mitochondrion were deposited in GenBank under the accession numbers KF554427 and KF554428, respectively.

For comparison of the mt genomes of ArM0029B, we used all four mt genomes to date reported in Trebouxiophyceae: *Prototheca wickerhamii* (NC_001613), *Helicosporidium* sp. ex Simulium jonesi (NC_017841), *Coccomyxa sp*. C-169 (NC_015316), and Trebouxiophyceae sp. MX-AZ01 (NC_018568). We skipped *Parachlorella minor* because of the very low gene content and arguable placement to be included in Trebouxiophyceae. For the plastid genomes of ArM0029B, we selected four reported species, including *C. variabilis* NC64A (NC_015359), *C. vulgaris* C-27 (NC_001865), *Coccomyxa* sp. C-169 (NC_015084), and *Trebouxiophyceae* sp. MX-AZ01 (NC_018569). We did not include non-photosynthetic Trebouxiophyceae species to compare plastid genomes because they do not contain many photosynthetic genes.

### Comparative analysis of cp genomes

The complete cp genomes of ArM0029B, *C. variabilis* NC64A, and *C. vulgaris* C-27 were compared using the MAUVE alignment tool [38] to identify rearrangement-free LCBs (locally collinear blocks) among genomes, yielding 25 LCBs with a minimum weight of 170. The genome sequence of the *C. variabilis* chloroplast was artificially rearranged prior to the MAUVE alignment so that the genome-level alignments could be maximally shown. Conserved genes among the three cp genomes were identified using the BLASTN search. genoPlotR [39] was then used to visualize conserved genes in the context of genomes and LCBs.

### Secondary structure analyses of intron and mt trn genes

The secondary structure of the group I intron was constructed based on the methods of Burke *et al.*[40] and Michel and Westhoff [41]. For the secondary structure of mt trn genes, the method of Chuang *et al.*[42] was consulted.

### Phylogenetic analyses

The phylogenetic relationships of ArM0029B among green algae were investigated using both chloroplast and mitochondrion genomic information. As an ingroup, all reported trebouxiophycean organellar genomic information was included. Currently, information concerning six cp genomes is available in Trebouxiophyceae: *Chlorella vulgaris* C-27 (AB_001684.1), *Chlorella variabilis* NC64A (NC_015359), *Parachlorella kessleri* (NC_012978), *Coccomyxa* sp. C-169 (NC_015084), *Oocystis solitaria* (FJ968739), and Trebouxiophyceae sp. MX-AZ01 (NC_018569). Four reported trebouxiophycean algal mt genomes are *Coccomyxa* sp. C-169 (NC_015316), *Helicosporidium sp*. ex Simulium jonesi (NC_017841), *Prototheca wickerhamii* (NC_001613), and Trebouxiophyceae sp. MX-AZ01 (NC_018568). A partial clone (AB011523) of the *cox1* gene for *Chlorella vulgaris* was also used. To avoid bias by taxon sampling, four chlorophycean algae, known both as cp and mt genomes, were used as an outgroup. These include *Chlamydomonas reinardtii* (NC_005353 for cp; NC_001638 for mt), *Gonium pectoral* (AP_012494 for cp; NC_020437 for mt), *Dunaliella salina* (GQ_250046 for cp; NC_012930 for mt), and *Scenedesmus obliquus* (DQ_396875 for cp; X17375 for mt).

DNA sequences of seven cp protein genes, including *psaA*, *psaB*, *psbA*, *psbB*, *psbC*, *psbD*, and *rbcL* were used for the cp phylogenetic MP, NJ and ML tree using Paup ver. 6.0. Bootstrap and jackknife analyses of MP tree were also performed with 1,000 replication. Shared gene contents among chlorophycean and trebouxiophycean algal mitochondria were very limited, and the DNA sequence variation of protein-coding genes was highly variable for successful alignment. Translated amino acid sequences of seven protein-coding genes, including *cob*, *cox1*, *nad1*, *nad2*, *nad4*, *nad5*, and *nad6* were used for the mt MP tree. In the analysis, gapped sequences were not included. Bootstrap and jackknife analyses were also performed with 1,000 replication.

## Competing interests

The authors declare that they have no competing interests.

## Authors’ contributions

W-JJ and JL designed the research and wrote the paper. HJ, JML, JP, YMS, and H-GC performed the research. All of the authors read and approved the manuscript.

## Supplementary Material

Additional file 1: Table S1 Additional putative CDSs predicted by Glimmer ver. 3.02. Additional 71 ORFs (>50 aa) were identified from the cp genome of ArM0029B using the Glimmer gene prediction tool. These ORFs were not added to the finalized gene set because *ab initio* gene prediction that uses a short-sized genome for self-training and prediction itself could result in many false genes. ORFs overlapping tRNA or rRNA genes were excluded. Click here for file

Additional file 2: Figure S1 Confirmation of a 15-kb gene cluster inversion in the plastid genome of *Chlorella sp*. ArM0028B compared with *C. variabilis* NC64A. (A) Diagram of the inverted gene cluster in the plastid genomes of *C. variabilis* NC64A and *Chlorella* sp. ArM0029B. PCR primers are marked as psaBF, ycf20R, psbAF, and rps14R with arrows. (B) PCR confirmation of a 15-kb gene cluster inversion in the plastid genome of *Chlorella sp*. ArM0028B. Lane 1: primer set (psaBF and ycf20R); Lane 2: primer set (psbAF and rps14R); Lane 3: primer set (psaBF and psbAF); Lane 4, primer set (ycf20R and rps14R). The expected sizes of PCR products in lanes 1 and 2 are 801 bp and 855 bp, respectively. The primers sequences used for PCR are follows. 5′-TATGTTTTAACTTATGCGGCATTCTT-3′ for psaBF; 5′-AACATTGAATTGCAAAAATGTTCC-3′ for ycf20R; 5′-CAACCGATGTATAAACGGTTTTCA-3′ for psbAF; 5′-TCTTCAAGGTCTTTTACCTGGT-3′ for rps14R. Total genomic DNA purified from ARM0029B was used for PCR reactions. PCR amplifications in only lanes 1 and 2 with the expected sizes indicating that a 15-kb gene cluster in the plastid genome of ArM0029B exists in the inverse orientation compared with the plastid genome of *C. variabilis* NC64A. Click here for file

Additional file 3: Figure S2 Conserved plastid genome gene clusters among trebouxiophycean algae. The arrangement of cp genes, including trnC to rpoC2 and rps14, are compared in ArM0029B and related species. The red-boldface indicates genes with a conserved order. The direction of the box arrow denotes sense orientation of transcription of the gene. Click here for file

Additional file 4: Figure S3 Secondary structure of mt trn genes in *Chlorella sp*. ArM0029B. Click here for file

Additional file 5: Figure S4 Single ML (A, HYK85 + G + I model) and NJ (B) trees from the DNA sequences of seven cp genes and a NJ tree (C) from translated amino acid sequences of seven mt protein-coding genes. */*: 100% bootstrap support/100% jackknife support. −/−: bootstrap and jackknife not supported. Click here for file

Additional file 6: Table S2. Distance matrix of seven cp gene sequences in Trebouxiophyceae. Click here for file
