# Supplementary material for: Plastid and mitochondrion genomic sequences from Arctic Chlorella sp. ArM0029B
Source: BMC Genomics. 2014 Apr 16;15:286. doi: 10.1186/1471-2164-15-286 (PMC4023601; doi:10.1186/1471-2164-15-286)

Additional Figure S1.

A

C. variabilis NC64A psaB-**psbA-ycf1-cysT-rpl32-ccsA-chlL-chlN-trnR1-minD-trnN-psaC-ycf20-trnI**(CAU)-rps14

Chlorella sp ArM0029B psaB-**trnI**(CAU)-**ycf20-psaC-trnN-minD-trnR1-chlN-chlL-ccsA-rpl32-cysT-ycf1-psbA**-rps14

→ ← → ←

B

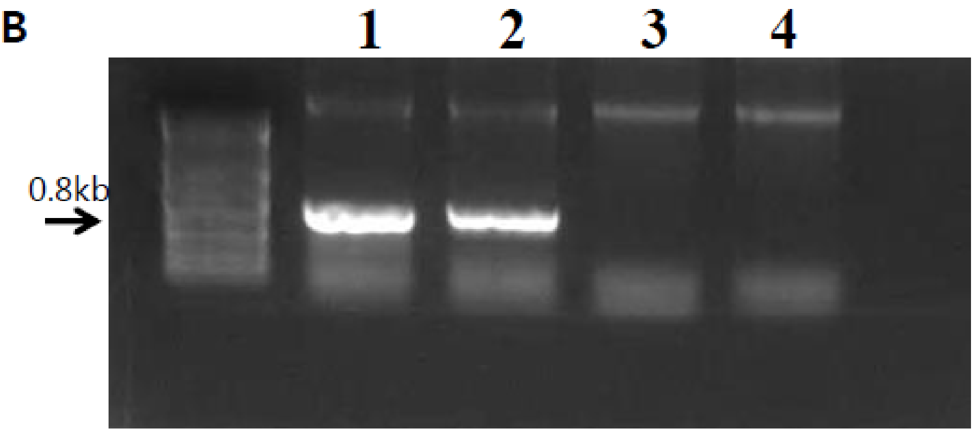

Supplement: Additional file 2: Figure S1 — Confirmation of a 15-kb gene cluster inversion in the plastid genome of Chlorella sp. ArM0028B compared with C. variabilis NC64A. (A) Diagram of the inverted gene cluster in the plastid genomes of C. variabilis NC64A and Chlorella sp. ArM0029B. PCR primers are marked as psaBF, ycf20R, psbAF, and rps14R with arrows. (B) PCR confirmation of a 15-kb gene cluster inversion in the plastid genome of Chlorella sp. ArM0028B. Lane 1: primer set (psaBF and ycf20R); Lane 2: primer set (psbAF and rps14R); Lane 3: primer set (psaBF and psbAF); Lane 4, primer set (ycf20R and rps14R). The expected sizes of PCR products in lanes 1 and 2 are 801 bp and 855 bp, respectively. The primers sequences used for PCR are follows. 5′-TATGTTTTAACTTATGCGGCATTCTT-3′ for psaBF; 5′-AACATTGAATTGCAAAAATGTTCC-3′ for ycf20R; 5′-CAACCGATGTATAAACGGTTTTCA-3′ for psbAF; 5′-TCTTCAAGGTCTTTTACCTGGT-3′ for rps14R. Total genomic DNA purified from ARM0029B was used for PCR reactions. PCR amplifications in only lanes 1 and 2 with the expected sizes indicating that a 15-kb gene cluster in the plastid genome of ArM0029B exists in the inverse orientation compared with the plastid genome of C. variabilis NC64A. [file 1471-2164-15-286-S2.pdf]
