# Supplementary material for: Plastid and mitochondrion genomic sequences from Arctic Chlorella sp. ArM0029B
Source: BMC Genomics. 2014 Apr 16;15:286. doi: 10.1186/1471-2164-15-286 (PMC4023601; doi:10.1186/1471-2164-15-286)

Additional Figure S2.

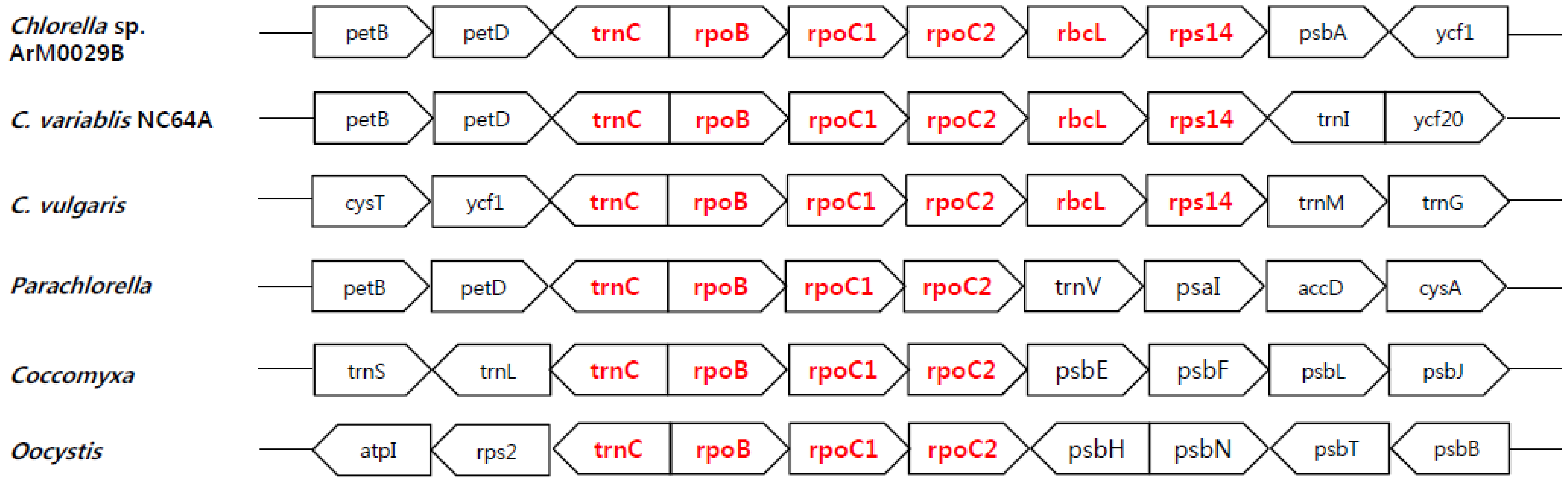

Supplement: Additional file 3: Figure S2 — Conserved plastid genome gene clusters among trebouxiophycean algae. The arrangement of cp genes, including trnC to rpoC2 and rps14, are compared in ArM0029B and related species. The red-boldface indicates genes with a conserved order. The direction of the box arrow denotes sense orientation of transcription of the gene. [file 1471-2164-15-286-S3.pdf]
