# Supplementary figures and images for: Plastid and mitochondrion genomic sequences from Arctic Chlorella sp. ArM0029B
Source: BMC Genomics. 2014 Apr 16;15:286. doi: 10.1186/1471-2164-15-286 (PMC4023601; doi:10.1186/1471-2164-15-286)

Additional Figure S3.

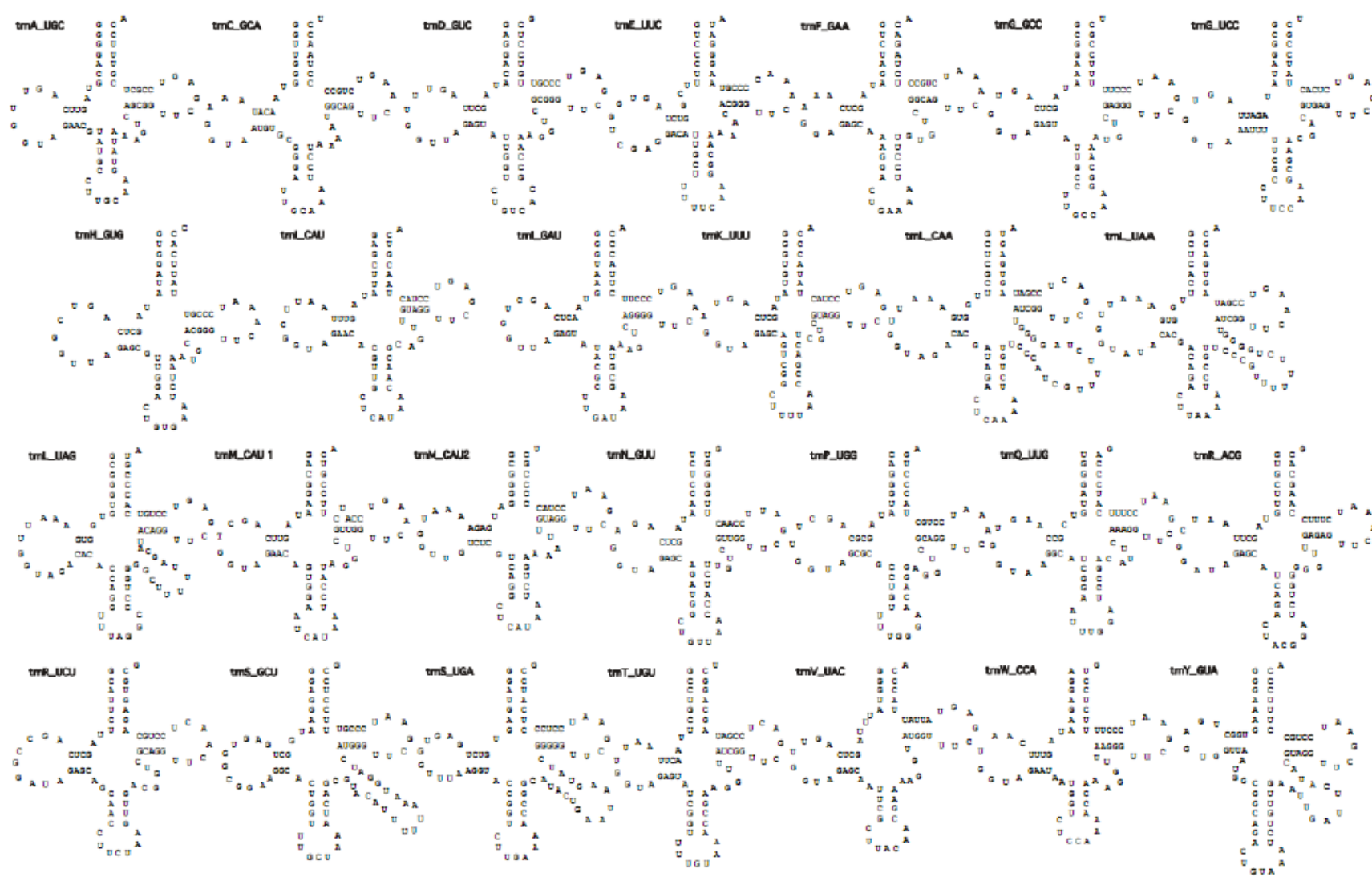

Supplement: Additional file 4: Figure S3 — Secondary structure of mt trn genes in Chlorella sp. ArM0029B. [file 1471-2164-15-286-S4.pdf]

Additional Figure S4.

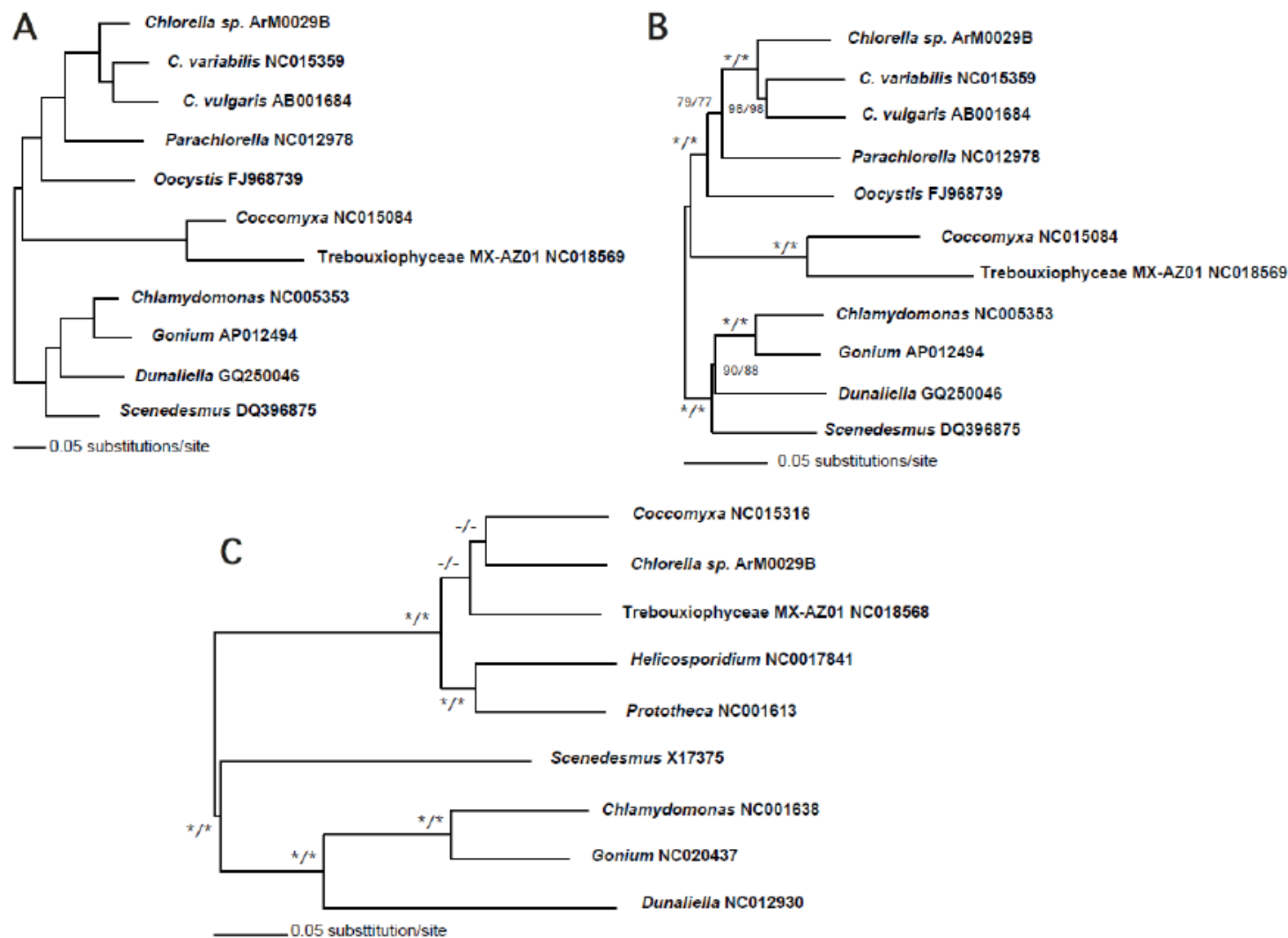

Supplement: Additional file 5: Figure S4 — Single ML (A, HYK85 + G + I model) and NJ (B) trees from the DNA sequences of seven cp genes and a NJ tree (C) from translated amino acid sequences of seven mt protein-coding genes. */*: 100% bootstrap support/100% jackknife support. −/−: bootstrap and jackknife not supported. [file 1471-2164-15-286-S5.pdf]
