# Supplementary material for: Plastid and mitochondrion genomic sequences from Arctic Chlorella sp. ArM0029B
Source: BMC Genomics. 2014 Apr 16;15:286. doi: 10.1186/1471-2164-15-286 (PMC4023601; doi:10.1186/1471-2164-15-286)
Supplement: Additional file 6: Table S2. — Distance matrix of seven cp gene sequences in Trebouxiophyceae. [file 1471-2164-15-286-S6.pdf]

Additional Table S2.

Taxa	1	2	3	4	5	6	7
1. ArM0029B	_						
2. C. variabilis	0.09193	_					
3. C. vulgaris	0.10299	0.09403	_				
4. Parachlorella	0.14071	0.14585	0.14328	_			
5. Oocystis	0.15911	0.16435	0.16287	0.14959	_		
6. Coccomyxa	0.22321	0.22932	0.22689	0.23112	0.22422	_	
7. MX-AZ01	0.25442	0.25684	0.25652	0.26705	0.2589	0.16901	_
